# Supplementary material for: Prognostic Value of Optic Nerve Sheath Diameters after Acute Ischemic Stroke According to Slice Thickness on Computed Tomography
Source: Diagnostics (Basel). 2024 Aug 12;14(16):1754. doi: 10.3390/diagnostics14161754 (PMC11354098; doi:10.3390/diagnostics14161754)
Supplement: Supplementary file 1 [file diagnostics-14-01754-s001.zip › diagnostics-3136391-suppl.pdf]

Supplementary Materials

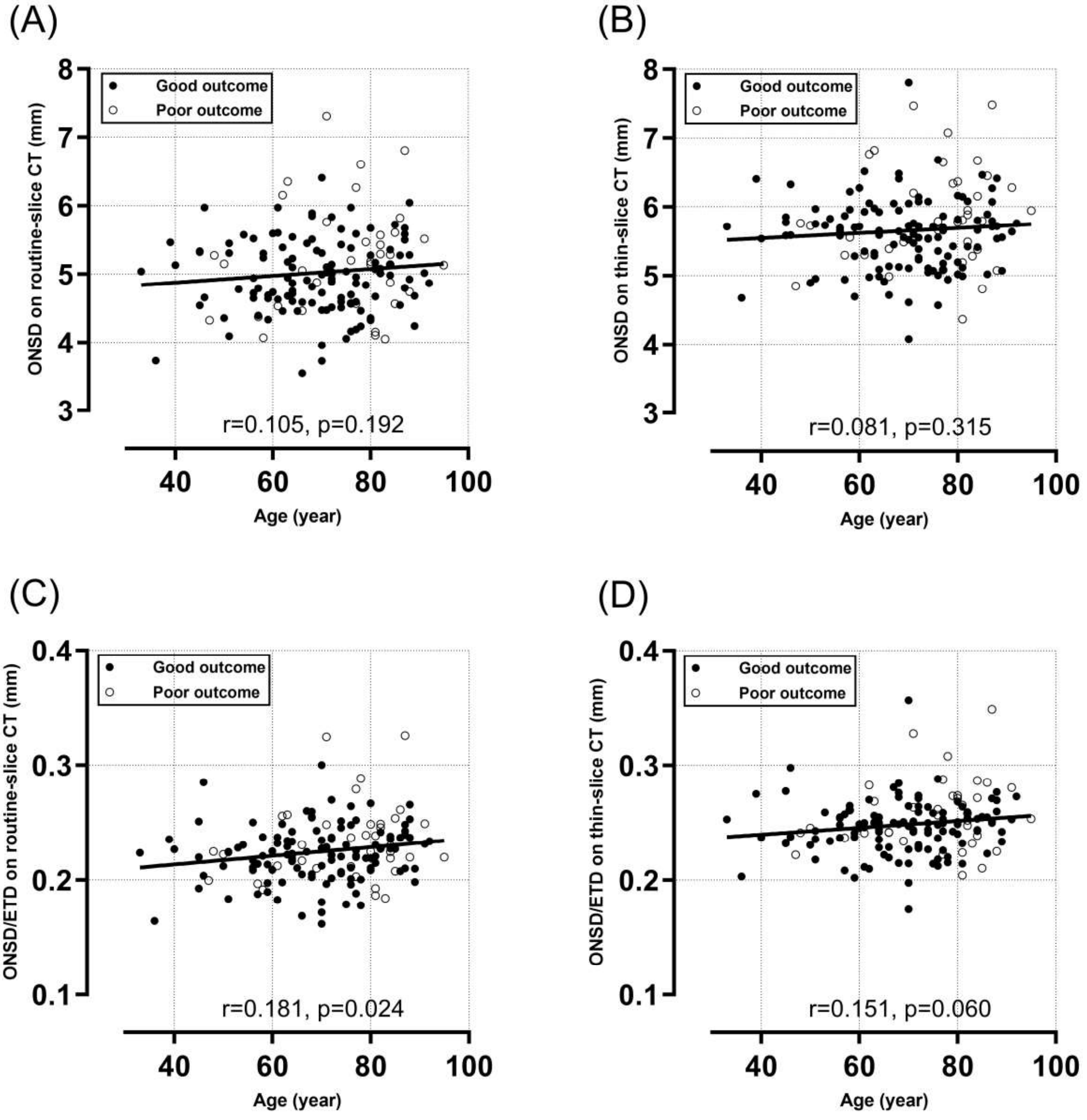

Figure S1. The correlations between age and ONSD-related variables (routine-slice ONSD [A], thin-slice ONSD [B], routine-slice ONSD/ETD [C] and thin ETD/ONSD [D]).

The Pearson correlation coefficients (r) and p values are indicated.

ONSD, optic nerve sheath diameter; CT, computed tomography; ETD, eyeball transverse diameter.

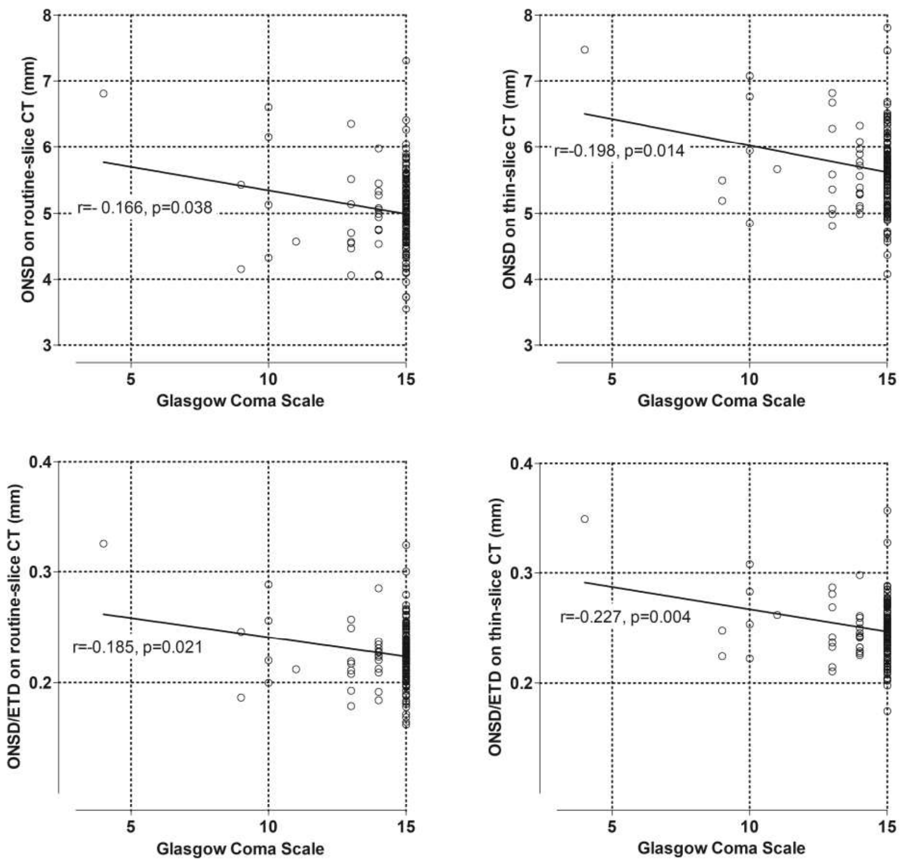

Figure S2. The correlations between the Glasgow Coma Scale score and ONSD-related variables. The Pearson correlation coefficients (r) and p values are indicated.

Table S1. Interrater reliability between two of the investigators.

Measures	Coefficient (95% confidence interval)
Routine-slice ONSD	0.960 (0.946–0.971)
Thin-slice ONSD	0.957 (0.940–0.968)
Routine-slice ONSD/ETD	0.944 (0.923–0.959)
Thin-slice ONSD/ETD	0.937 (0.914–0.954)
